# Moss survival through *in situ* cryptobiosis after six centuries of glacier burial

**DOI:** 10.1038/s41598-017-04848-6

**Published:** 2017-06-30

**Authors:** N. Cannone, T. Corinti, F. Malfasi, P. Gerola, A. Vianelli, I. Vanetti, S. Zaccara, P. Convey, M. Guglielmin

**Affiliations:** 1 Dept. Sciences and High Technology, Insubria University, Via Valleggio, 11 – 22100 Como (CO) Italy; 2 Dept. Theoretical and Applied Sciences, Insubria University, Via Dunant, 3 – 21100 Varese (VA) Italy; 3 British Antarctic Survey, Natural Environment Research Council, High Cross, Madingley Road, Cambridge, CB3 0ET United Kingdom

## Abstract

Cryptobiosis is a reversible ametabolic state of life characterized by the ceasing of all metabolic processes, allowing survival of periods of intense adverse conditions. Here we show that 1) entire moss individuals, dated by ^14^C, survived through cryptobiosis during six centuries of cold-based glacier burial in Antarctica, 2) after re-exposure due to glacier retreat, instead of dying (due to high rates of respiration supporting repair processes), at least some of these mosses were able to return to a metabolically active state and remain alive. Moss survival was assessed through growth experiments and, for the first time, through vitality measurements. Future investigations on the genetic pathways involved in cryptobiosis and the subsequent recovery mechanisms will provide key information on their applicability to other systematic groups, with implications for fields as divergent as medicine, biodiversity conservation, agriculture and space exploration.

## Introduction

Cryptobiosis is a reversible ametabolic state of life that has been considered as a third state between life and death^1^. It is characterized by the ceasing of all metabolic processes and allows survival of periods of intense adverse environmental conditions such as desiccation, freezing or oxygen deficiency, or combinations of these^1, 2^. The occurrence of cryptobiosis is documented in several invertebrate groups including rotifers, chironomid midges, nematodes and tardigrades^1, 3^, as well as in many microbial groups occurring in polar and other extreme environments^4, 5^. Cryptobiosis differs from dormancy (hibernation, aestivation, diapause or quiescence), which is associated with a low but still measurable level of metabolism (or hypometabolism), albeit with the temporary cessation of growth, development and (in animals) physical activity^2^. Dormancy is well documented in the invertebrates, plants and microbes of extreme environments^6^, allowing their survival during inclement periods through different mechanisms (e.g. tolerance of freezing, desiccation).

Cryptobiosis is a survival strategy that appears particularly suited to the permafrost environment. Several different microbial groups are known to remain viable in permafrost, surviving under the permanently frozen habitat conditions possibly from the point of their formation, in some cases up to 30,000 years^4, 7^. At subzero temperatures the rates of biochemical reactions and processes become extremely low, and ensure the preservation of the biological system^4^.

Survival under frozen or anhydrobiotic conditions in nematodes, rotifers and tardigrades, widely acknowledged to be three of the most resistant invertebrate groups, has likewise been demonstrated over periods of only a decade or two at most^7, 8^. Survival through cryptobiosis in mosses and lichens has been reported for laboratory experiments by Keilin^2^: fragments of mosses and lichens previously desiccated and cooled at −273 °C revived after being kept for about 2 hours at −272 °C. Direct regeneration after cryptobiosis has been demonstrated in mosses in laboratory experiments from herbarium (dried) and frozen material preserved for 20 years at most^9, 10^ and over shorter periods (five years) for ferns and angiosperms^11^. Long-term cryoptobiosis lasting for centuries has been described for angiosperm seeds, although seed germination was obtained only in the laboratory through cloning and *in vitro* culturing approaches^4, 12, 13^. Regeneration, suggested by *in vivo* field observations, was recently demonstrated through *in vitro* growth from exhumed mosses re-exposed by glacier retreat after 400 years of burial by a cold-based glacier in Canada, demonstrating the totipotent capacity of bryophytes^14^. Millennial scale viability has been described from a core of an *in situ* moss bank in Antarctica preserved within permafrost that, after thawing in the laboratory, showed regeneration of moss shoots^15^. These studies were interpreted as cases of cryptobiosis, but did not involve direct measurements of moss metabolic vitality.

Mosses are major components of plant communities in terms of diversity and biomass in high latitude ecosystems in the High Arctic and in Antarctica, where macroscopic vegetation is dominated by cryptogams^16, 17^. Mosses are poikilohydric organisms with high degree of phenotypic plasticity^18^ and have well-developed features allowing them to tolerate stress^15^ and thrive in environments subject to change and disturbance^16^. Among the key traits allowing mosses to persist in harsh environments, one of the most important is tolerance to desiccation and drought^18^, also contributing to their ability to resist freezing (as during the Antarctic winter)^19^.

In this study, measurements of metabolic activity through chlorophyll *a* fluorescence (as indicators of vitality and survival) and growth chamber experiments applied to mosses exposed by recent glacier retreat at Rothera Point (maritime Antarctica) present evidence of: a) *in situ* moss survival for over more than 600 y in the natural habitat through cryptobiosis, and their subsequent spontaneous recovery to active metabolism after glacier retreat, b) persistence of active metabolism and growth in surviving exhumed mosses, c) regeneration with development of green stem apices from moribund stems of exhumed mosses.

## Results

### Direct Observations and Radiocarbon Dating

**Figure 1:**
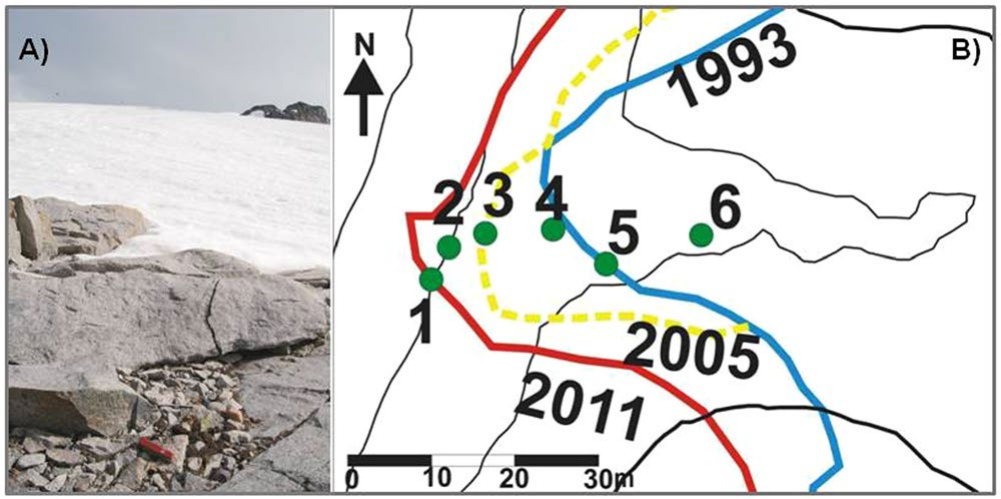
(**A**) View of the glacial margin of the Wormald Ice Piedmont (Rothera Point, Antarctic Peninsula, taken in 2009). The occurrence of exhumed patches of the moss *Bryum pseudotriquetrum* can be seen in the foreground. (**B**) Map showing the location of the moss samples (see Table 1) with the position of the glacier boundary in 1993 (blue line), 2005 (yellow line) and 2011 (red line) (map created by CorelDraw 6.0).

Two moss species, *Bryum pseudotriquetrum* (Hedw.) P. Gaertn. and *Sanionia uncinata* (Hedw.) Loeske, were collected from six sites in the glacier front (Fig. 1A,B) and foreland: five samples of exhumed mosses (n. 1–5), plus an extant moss (n. 6, located at 16.8 m from the ice margin in 2011) used for comparison (Table 1). The sample n. 1 was emergent during the week of the collection, and the sample n. 2 was located less than 1 m from the glacier front and likely exhumed less than one year previously (Table 1). The remaining three samples (n. 3–5, Table 1) were located close to the glacier front (between <1 m and 10.1 m from the ice margin in 2011) and exhumed between six and 18 years ago (Table 1). All exhumed samples were from *in situ* habitats in the field, with exceptional preservation and no damage apparent to the gametophytes which exhibited intact rhizoids, stems and leaves. No sporophyte tissue was present. All samples lacked any sign of regrowth, with green lateral branches and stem apices being absent. The calibrated age (see methods) of three of the most recently exhumed moss (n. 1, 3, 4) was determined by radiocarbon dating and ranged between 640–526 and 626–510 cal years BP (Table 1).

**Table 1:** Moss samples collected along the boundary of the Wormald Ice Piedmont from the ice/glacier foreland, presenting conventional and calibrated age 2σ range (cal y BP); maximum time since exhumation after glacier retreat (see Fig. 1B); minimum distance from the ice margin (m) (DIM); species name; visual observations (field and dissecting microscope prior the 2014 regrowth experiment); value of Fv/Fm (variable fluorescence/maximum fluorescence) of the chlorophyll *a* fluorescence measurements; shape of the induction curve obtained during chlorophyll *a* fluorescence measurements; response during the growth experiment.

Sample #	Lab code for 14 C dating (Beta)	Conventional Age BP	Calibrated age 2σ range (cal y BP)	Maximum time since exhumation (yr); DIM (m)	Species name	Visual Observations	Chlorophyll *a* measurement of metabolic activity (Fv/Fm)	Induction curve shape	Response during growth experiment
1	356172	580 ± 30	626–510	0 y; at ice margin	SU	Active	0.39 ± 0.10	Active	Alive+Growth
2			626–510	1 y; <1 m	BP	Active	0.49 ± 0.13	Active	Alive
3	457644	600 ± 30	631–519	6 y; 6 m	BP	Moribund	0.28 ± 0.07	Inactive	Moribund
4	457643	620 ± 30	640–526	18 y; 9.7 m	BP	Moribund	0.28 ± 0.10	Inactive	Regeneration
5				18 y; 10.1 m	BP	Moribund	0.29 ± 0.04	Inactive	Regeneration
6			Extant	Never glacier covered; 16.8 m	BP	Active	0.53 ± 0.11	Active	Alive+Growth

Legend: BP = *Bryum pseudotriquetrum*; SU = *Sanionia uncinata*.

**Figure 2:**
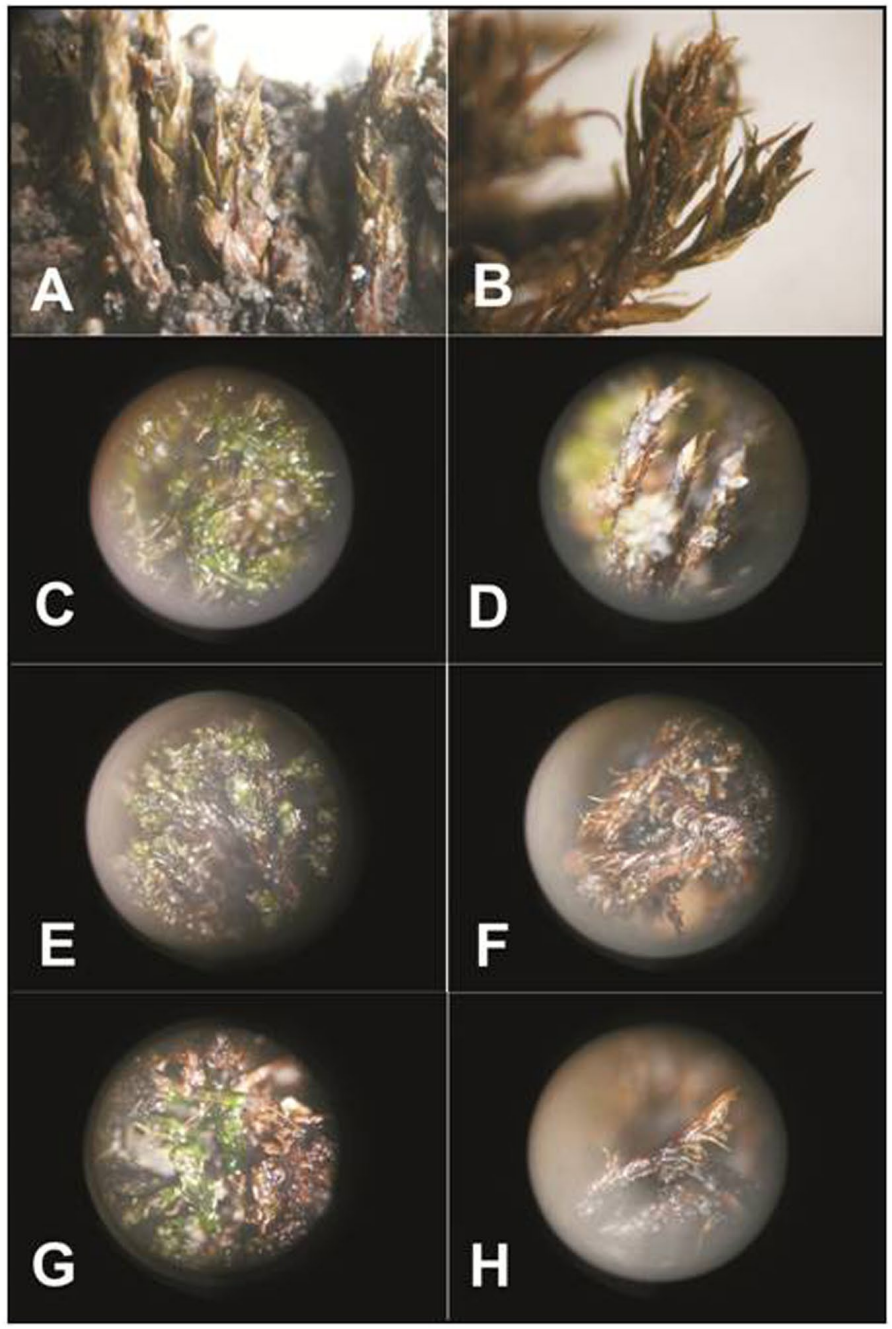
Moss samples of *Bryum pseudotriquetrum* (Hedw.) P. Gaertn. (**A**–**E**,**G**) and *Sanionia uncinata* (Hedw.) Loeske (**F**,**H**) that were assessed as active in the field ((**A**,**B**) sample n. 2), remained alive and exhibited regeneration during the growth experiment ((**C**–**E**,**G**) samples n. 4, 5 (**F**,**H**) sample n. 1).

Based on field observations and examination in the laboratory with a dissecting microscope (after sample collection, and prior to the growth experiment), two exhumed mosses (n. 1, 2) and the extant sample (n. 6) showed persistence of visibly greenish material with green leaves (Fig. 2A,B) lacking any decomposition processes and/or development of moulds. The other three exhumed samples (n. 3–5) were characterized by blackish leaves, and/or their desiccation (Table 1). From these observations, we suggest the two recently exhumed and extant mosses were biologically active populations whereas the other three exhumed mosses were moribund (Table 1).

### Vitality Measurements

**Figure 3:**
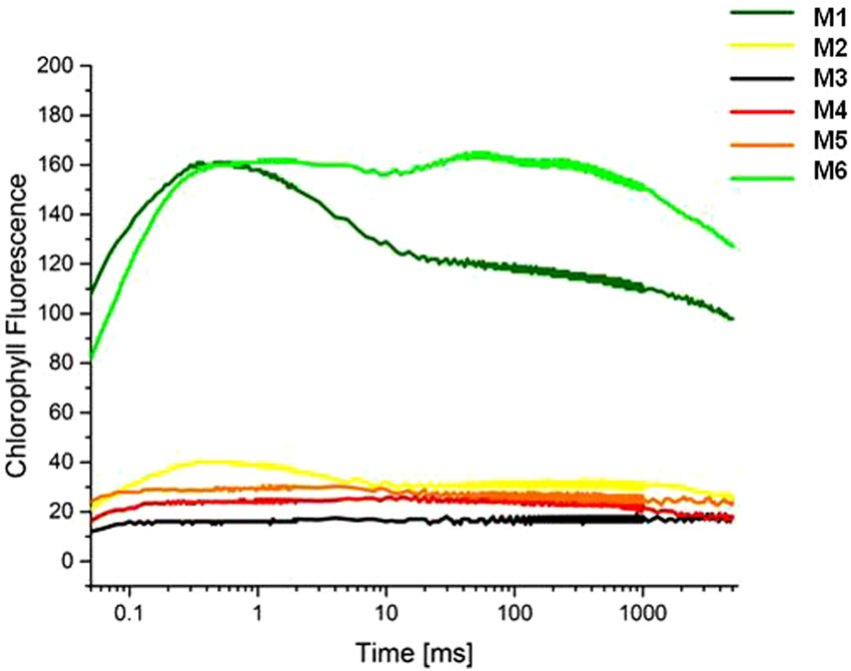
Fluorescence induction curves indicating the kinetics of the light reaction of the six moss samples. The Y axis indicates the chlorophyll fluorescence emission as radiometric quantification. Legend: M1-M6 indicate the sample numbers (1–6) as reported in Table 1.

Moss vitality was tested by analyzing the metabolic activity of the gametophores using Chlorophyll *a* fluorescence with reference to a) the values of Fv/Fm (variable fluorescence/maximum fluorescence maximum) (Table 1), and b) the shape of the induction curve (Fig. 3). We considered the gametophytes to be metabolically active when Fv/Fm >0.2^20, 21^. Applying this threshold, metabolic activity (compatible with life/vitality) was detected for all the three mosses suggested to be biologically active based on visual inspection (n. 1, 2, 6). The three mosses suggested to be moribund (n. 3–5) exhibited values of Fv/Fm slightly higher than the threshold of 0.2, also indicating metabolic activity, but at a poor level.

Analyzing the fluorescence induction curve shape, the progressive flattening observed comparing the extant moss specimen with the two recently exposed moss clumps indicated a progressive decrease of activity. Conversely, the flattened shape of the fluorescence induction curve of the three mosses suggested them to be moribund (samples n. 3–5) and indicated a lack of activity.

### Growth Experiment

During the growth experiment, the three metabolically active samples (based on their Fv/Fm values; n. 1, 2, 6) remained alive (sample n. 2, Fig. 2A,B), with the extant moss (n. 6) and the recently exposed sample of *S. uncinata* (n. 1) exhibiting growth (Table 1, Fig. 2F,H), and growth occurred from the stem apex in both the latter cases (without any protonemal or rhizoid initial growth). Among the moribund mosses, sample n. 3 became marcescent and covered by fungal hyphae, and did not show any regeneration (Table 1), whereas the other two moribund samples (n. 4, 5) exhibited regeneration (Fig. 2C–E,G), with growth of leaves at the branch apices in existing gametophyte shoots (Table 1).

## Discussion

Cryptobiosis has already been demonstrated for mosses in polar environments, based on *in vivo* observations^14^ and *in vitro* regeneration experiments^14, 15^. Our data, based on fluorescence measurements, corroborate these previous studies. Cryptobiosis in our study is indicated by long-term survival for six centuries, but the study’s true novelty is the demonstration, based on vitality measurements as well as on growth experiments, of the ability of entire moss individuals to withstand six centuries of cryptobiosis and, at its cessation, to spontaneously recover to active metabolism and survive *in situ* following glacier retreat. Indeed, the chlorophyll *a* fluorescence measurements indicated the occurrence of metabolic activity (compatible with life) for all the three mosses hypothesized to be active based on visual inspection (n. 1, 2, 6). Their Fv/Fm values were lower than would typically be recorded from extant field samples (ranging between 0.6 and 0.8)^21^. However, it is well known that repeated freeze-thaw cycles decrease Fv/Fm, especially when mosses are exposed to light^22^, as would be the case for these mosses re-exposed by the glacier retreat during the austral summer (with 24 hours of light per day). These results are confirmed by the fluorescence induction curve (Fig. 3), that shows a decrease of activity from the extant moss and the two recently exposed mosses. This is similar to the report of Rutten and Santarius^23^ when testing age-related differences in frost tolerance of mosses subject to experimental freezing, in particular, the data obtained from the extant moss (sample n. 6, Fig. 3 curve M6) with young leaves (cf. Rutten and Santarius, Fig. 3 curve 11), the most recently exhumed moss (sample n. 1, Fig. 3 curve M1) with mature leaves (cf. Rutten and Santarius, Fig. 3 curve 10), and the 1 y exhumed moss (sample n. 2, Fig. 3 curve M2) with old leaves (cf. Rutten and Santarius, Fig. 3 curve 6).

All vitality analyses indicated, at best, poor metabolic activity for the mosses with longer re-exposure times and suggested to be moribund on the basis of visual observations (n. 3–5). Using the extant moss specimen for comparison, our data indicated the persistence of active metabolism and capacity for regrowth of the very recently re-exposed mosses (n. 1), whereas survival and active metabolism were observed for the less recently exhumed moss clumps (≤1 y) (sample n. 2). The mosses that had been exposed for longer intervals were considered moribund as indicated by poor or lack of metabolic activity, but showed regeneration in the growth experiment, as noted in some previous studies^14, 15^.

Recovery processes leading to resumption of normal metabolic activity after cryptobiosis are often characterized by high rates of respiration repair processes that, in some cases, can be lethal^24^, especially if mosses are exposed to light, due to photoinhibitory damage^22^. The intensity and frequency of these recovery processes may explain the different ability of mosses to survive and grow after different periods of re-exposure. As time progresses (>1 y) after re-exposure, the mosses will face repeated seasonal dormancy and adverse conditions^25^. Therefore, accumulated damage with the recovery processes will increase with time, explaining why longer re-exposure time may be associated with the observed lower vitality.

Future perspectives may concern investigations on the genetic mechanisms involved in cryptobiosis and the subsequent recovery mechanisms, which would also provide information on the potential exportability and applicability of these mechanisms to other systematic groups (including humans), with potential relevance for medicine (e.g. organ banking through the cryopreservation of complex tissues^26^), biodiversity conservation (germplasm, embryogenic cells of both gymnosperms and angiosperms^27^), agriculture (cultivars of major food crops^28^) and space exploration (dormancy during space travel^29^, use of anhydrobiosis and cryobiosis to protect organisms exposed to space and exoplanetary environments^30^).

## Materials and Methods

### Study area

The study area was located at Rothera Point (67°34′S; 68°07′W) on Adelaide Island, Marguerite Bay, southern Maritime Antarctic. The climate is a cold dry maritime climate, with mean annual air temperature of −4.2 °C and mean annual precipitation of about 500 mm, of which 20% occurs as rain. Permafrost is continuous and exceeds 100 m in depth with an active layer ranging between 0.76 and 1.40 m. Vegetation on Rothera Point is scattered and mainly composed of epilithic lichens (dominated by *Usnea sphacelata* and *Umbilicaria decussata*), and sporadic mosses.

The study site was located at the southern limit of the Wormald Ice Piedmont Glacier, locally known as the “ice ramp”, and located between 10 and 110 m a.s.l. This glacier front has been monitored since 1989 and has receded rapidly in recent decades, with ablation rates (surface lowering) of 0.32 m water equivalent between 1989 and 1997, and a frontal retreat that increased from 0.85 m/y (period 1997–2005) to 1 m/y (2005–2011).

### Field sampling

In February 2009 some scattered moss patches with 1–2 cm of underlying organic matter were observed among the boulders at 1.5 m from the 2009 ice/glacier front. These were sampled for radiocarbon dating and used in the reconstruction of the glacier’s dynamics since the Medieval Warm Period^31^. In January 2011 the glacier foreland was re-sampled at distances between <1 m and 16. 8 m to the then existing glacier front, collecting six samples used for the present study (Table 1): five samples of exhumed mosses (n. 1: *Sanionia uncinata* (Hedw.) Loeske; n. 2–5: *Bryum pseudotriquetrum* (Hedw.) P. Gaertn.); plus n.6: an extant sample of *B. pseudotriquetrum* observed in the glacier foreland at a larger distance from the glacier front and used as reference for comparison. The moss samples were identified in the laboratory following Ochyra *et al*.^17^ and observation under the dissecting microscope. The moss samples were immediately stored at −20 °C, transported to Italy and kept at −20 °C until experimental studies commenced in March 2014. Sub-samples of the five exhumed moss specimens and one extant specimen were assayed for chlorophyll *a* fluorescence measurements and a growth experiment (Table 1). Three samples (n. 1, 3, 4) collected in February 2011 (Table 1) were also subsampled for radiocarbon dating.

### ^14^C Dating

Age determination of samples followed a standard ^14^C protocol, as used also for the reconstruction of the glacier dynamics during the Medieval Warm Period^31^. The moss samples selected for radiocarbon dating (samples n. 1, 3, 4) were kept frozen between collection and being sent to Beta Analytic laboratories (Miami, USA). These, moss samples were subjected to an acid–alkali–acid pre-treatment according to Beta Analytic standard procedures. After pre-treatment, samples for radiocarbon dating were prepared for AMS by converting them into graphite. Calibrated ages were calculated with the software OxCal 4.2 using the SHCal13 ^14^C Southern Hemisphere atmosphere dataset. Radiocarbon age data are reported as conventional radiocarbon years BP (^14^C yr BP) ±σ and as calibrated age ranges with a 2σ error (95.4%) (cal. yr BP; relative to AD 1950).

### Chlorophyll a fluorescence test to assess moss vitality

Chlorophyll *a* fluorescence provides a measurement of the metabolic activity of the gametophytes^21, 32^, with tissue having an Fv/Fm (variable fluorescence/maximum fluorescence) of 0.2 or higher^20, 21^ considered to be active. To assess the vitality of the six moss specimens, chlorophyll *a* fluorescence measurements were carried out in the laboratory with a portable Plant Efficiency Analyzer (PEA, Hansatech Instrument Ltd., Norfolk, UK) fluorometer. Three sub-samples of each of the six frozen moss samples were placed in Petri dishes and placed at ambient temperature (20 °C). Once thawed, all sub-samples experienced 24 h of dark-adaptation and hydration with deionized water^19, 22^. From each sub-sample five leaves were randomly detached and each leaf was placed in a different clip with shooters. Assays were performed after an additional dark period of 15 min^22, 33^. Measurements were performed with a light intensity of about 3,000 mol m^−2^ s^−1^ provided by an array of six light-emitting diodes (peak wavelength at 650 nm) focused onto the sample to provide homogeneous illumination over the exposed area. Fluorescence values were recorded at different rates depending on the different phases of the induction kinetics, in order to provide the best time resolution. Initially data were sampled at 0.01 ms (millisecond) intervals for 2 ms (interval for initial data acquisition to determine both the minimum fluorescence value (F_*0*_) value as well as to monitor the initial rapid rise of the chlorophyll fluorescence signal); then slower acquisition rates of 1 ms and 100 ms were used for 2–1000 and 1000–5000 ms assay intervals, respectively, appropriate to follow the decrease of the kinetics of the fluorescence signal. Overall, for each 5 sec (second) assay 1258 data points were recorded. The *F*_0_ parameter (minimum fluorescence) represents the emission by excited chlorophyll *a* molecules in the antennae structure of Photosystem II (PSII) at time 0. Using a PEA fluorometer, *F*_0_ was calculated for the initial experimental data recorded at the onset of the illumination in the range of 80–120 μs; these data were analyzed using least squares regression by a linear function and then extrapolated to time 0, whose fluorescence signal is considered the *F*_0_ value^34^. The maximum fluorescence value (Fm) is obtained for a continuous light intensity, whereas Fv indicates the variable component of the recording, calculated by subtracting F_0_ from the Fm value. The maximum quantum efficiency of PSII was calculated according to the equation: Fv/Fm = (Fm − F_0_)/Fm^25, 35^.

The kinetics of the light reaction provides an assessment of the recovery of the electron transport in PSII by analyzing the shape of the fluorescence induction curve^35^. To identify all steps in the induction curve a logarithmic presentation of the time axis was used^34^. According to the standard procedure adopted when using PEA fluorometers, the time axis of the induction curve started from 50 μs^34^. Normally the induction curve is characterized by four steps (named O, J, I, P), however samples subject to freezing exhibited different sensitivity of young vs. mature vs. old leaves, with resultant changes of the induction curve shape^23^. To achieve further confirmation of the vitality of the samples analyzed, and to test for differences linked to their different exposure ages, the induction curves for each assay were recorded and logged.

### Growth experiment

The growth experiment utilized the natural substrate from beneath moss populations at Anchorage Island (close to Rothera Point) mixed with high quality commercial litter (Tercomposti s.r.l. Brescia). Twelve glass cups were filled each with 9 g of this soil substrate. The 12 filled cups were sterilized at 120 °C for 15 min, transferred into sterilized Petri dishes, sealed with parafilm and placed in a growth chamber (CV-36L5, Percival Scientifics Series 101) from 17 January 2014 to 17 March 2014. In the growth chamber they were kept under controlled conditions at a fixed temperature of 15 °C, with 16 h of light (17 W cool-white fluorescence lamps, light intensity of 64 µmoles/m^2^/s) and 8 h of darkness^14, 15^. After two months in the growth chamber all 12 Petri dishes with soil substratum were completely sterile. Six were then used for the moss samples and 6 as controls. On 18 March 2014 the moss samples were transferred into the Petri dishes with the soil substratum: in each Petri dish each moss sample was divided into three sub-samples. Then the 12 Petri dishes were returned to the growth chamber and maintained under the same controlled conditions^14, 15^. Once per week each Petri dish received 5 ml of deionized water (misting each specimen with a handheld spray bottle), which had previously been sterilized for 15 min at 120 °C^14^. All samples were placed in shallow trays and repositioned every 15 d to minimize effects of any variations in light intensity within the chamber^14^. The contents of the Petri dishes were inspected visually (including both the moss samples and the six soil control plots) and regularly observed under a dissecting microscope^15^ between 19 May and 2 December 2014.

## References

[1] Clegg JS (2001). Cryptobiosis-a peculiar state of biological organization. Comp. Biochem. Physiol.

[2] Keilin D (1959). The Problem of anabiosis or latent life: history and current concept. Proc. R. Soc. Series B.

[3] Neuman Y (2006). Cryptobiosis: a new theoretical perspective. Progr. Biophys. Molec. Biol.

[4] Yashina S (2012). Regeneration of whole fertile plants from 30,000-y-old fruit tissue buried in Siberian permafrost. PNAS.

[5] Graham DE (2012). Microbes in thawing permafrost: the unknown variable in the climate change equation. ISME J..

[6] Convey, P. *Life at Extremes* (ed. Bell, E.), Life in the cold: polar terrestrial, 81–102 (CABI Publishing, 2012).

[7] Rebecchi L, Altiero T, Guidetti R (2007). Anhydrobiosis: the extreme limit of desiccation tolerance. Invert. Survival J.

[8] Kagoshima H (2012). Multi-decadal survival of an Antarctic nematode, *Plectus murrayi*, in a −20 °C stored moss sample. Cryo-Lett..

[9] Longton RE, Holdgate MW (1967). Temperature relationships of Antarctic vegetation. Phil. Trans. Roy. Soc..

[10] Glime, J. M. *Bryophyte Ecology*. Vol. 1, Ch. 7–3, Table 1. Ebook sponsored by Michigan Technological University and the International Association of Bryologists http://www.bryoecol.mtu.edu/ (2007).

[11] Alpert P (2000). The discovery, scope, and puzzle of desiccation tolerance in plants. Pl. Ecol..

[12] Shen-Miller J (2002). Long-living lotus: Germination and soil γ-irradiation of centuries-old fruits, and cultivation, growth, and phenotypic abnormalities of offspring. Am J Bot.

[13] Sallon S (2008). Germination, genetics, and growth of an ancient date seed. Science.

[14] La Farge C, Williams KH, England JH (2013). Regeneration of Little Ice Age bryophytes emerging from a polar glacier with implications of totipotency in extreme environments. PNAS.

[15] Roads E, Longton RE, Convey P (2014). Millenial timescale regeneration in a moss from Antarctica. Curr. Biol..

[16] Longton, R. E. *The biology of polar bryophytes and lichens*. (Cambridge University Press, 1988).

[17] Ochyra, R., Bernadek-Ochyra, A. & Smith, R. I. L. *The illustrated moss flora of Antarctica*. (Cambridge University Press, 2008).

[18] Turetsky MR (2012). The resilience and functional role of moss in boreal and Arctic ecosystems. New Phytol..

[19] Schlensog M, Pannewitz S, Green GTA, Schroeter B (2004). Metabolic recovery of continental Antarctic cryptogams after winter. Polar Biol..

[20] Schroeter, B., Kappen, L., Green, T. G. A. & Seppelt, R. D. *Ecosystem processes in Antarctic ice-free landscapes* (eds Lyons, W. B., Howard-Williams, C., Hawes, I.), Lichens and the Antarctic environment: Effects of temperature and water availability on photosynthesis. pp. 103–117 (Balkema, 1997).

[21] Schlensog M, Green TGA, Schroeter B (2013). Life form and water source interact to determine active time and environment in cryptogams: an example from the maritime Antarctic. Oecologia.

[22] Lovelock CE, Jackson AE, Melick DR, Seppelt RD (1995). Reversible photoinhibition in Antarctic moss during freezing and thawing. Plant Physiol..

[23] Rutten D, Santarius KA (1992). Age-related differences in frost sensitivity of the photosynthetic apparatus of two *Plagiomnium* species. Planta.

[24] Proctor MC, Tuba Z (2002). Poikilohydry and homoihydry: Antithesis or spectrum of possibilities?. New Phytol..

[25] Schroeter B (2012). The moss *Bryum argenteum* var. *muticum* Brid. is well adapted to cope with high light in continental Antarctica. Ant. Sci..

[26] Lewis JK (2016). The Grand Challenges of Organ Banking: Proceedings from the first global summit on complex tissue cryopreservation. Cryobiology.

[27] Guan Y, Li S-G, Fan X-F, Su Z-H (2016). Application of somatic embryogenesis in woody plants. Front. Plant Sci..

[28] Uchendu EE, Shukla N, Saxena PK, Keller JER (2016). Cryopreservation of potato microtubers: the critical roles of sucrose and desiccation. Plant Cell Tiss. Organ. Cult..

[29] Cerri M (2016). Hibernation for space travel: Impact on radioprotection. Life Sci. Space Res..

[30] Guidetti R, Rizzo AM, Altiero T, Rebecchi L (2012). What can we learn from the toughest animals of the Earth? Water bears (tardigrades) as multicellular model organisms in order to perform scientific preparations for lunar exploration. Plan. Space Sci..

[31] Guglielmin M, Convey P, Malfasi M, Cannone N (2016). Glacial fluctuations since the ‘Medieval Warm Period’ at Rothera Point (western Antarctic Peninsula). Holocene.

[32] Proctor M (2001). Patterns of desiccation tolerance and recovery in bryophytes. Pl. Gr. Regulat.

[33] Lud D, Moerdjk TCW, van der Poll WH, Buma AGJ, Huisked AHL (2002). DNA damage and photosynthesis in Antarctic and Arctic *Sanionia uncinata* (Hedw.) Loeske under ambient and enhanced levels of UV-B radiation. Pl. Cell Env..

[34] Lazar D (2006). The polyphasic chlorophyll *a* fluorescence rise measured under high intensity of exciting light. Funct. Pl. Biol..

[35] Li Y (2010). Reorganization of photosystem II is involved in the rapid photosynthetic recovery of desert moss *Syntrichia caninervis* upon rehydration. J. Pl. Physiol..

